# A case of a thoracic duct cyst extending from the mediastinum to the cisterna chyli resected using bilateral thoracoscopic surgery in the prone position

**DOI:** 10.1186/s40792-023-01740-6

**Published:** 2023-09-25

**Authors:** Hironari Miyamoto, Shigeru Lee, Takemi Ishidate, Kenji Kuroda, Hiroaki Kasashima, Yuichiro Miki, Mami Yoshii, Tatsunari Fukuoka, Tatsuro Tamura, Masatsune Shibutani, Takahiro Toyokawa, Kiyoshi Maeda

**Affiliations:** Department of Gastroenterological Surgery, Osaka Metropolitan University Graduate School of Medicine, 1-4-3 Asahimachi, Abeno, Osaka 545-8585 Japan

**Keywords:** Thoracic duct cyst, Bilateral thoracoscopic surgery, Prone position

## Abstract

**Background:**

Thoracic duct cysts are extremely rare mediastinal tumors. We report a case of a thoracic duct cyst extending from the caudal aspect of the left main bronchus to the left renal artery that was safely and completely resected via bilateral thoracoscopic surgery in the prone position.

**Case presentation:**

A 77-year-old male was referred to our hospital for follow-up computed tomography (CT) of prostate cancer, which revealed a mediastinal tumor and fatty low-density along the posterior mediastinum of the para-aortic artery with a slightly high-density component. Magnetic resonance imaging revealed a T2-weighted image with high intensity. The preoperative radiological diagnosis was lipoma or well-differentiated liposarcoma. CT in the prone position suggested that the tumor could be resected from the thoracic cavity to the caudal side, and bilateral thoracoscopic surgery was performed in the prone position. Based on the surgical findings, the tumor was diagnosed as a thoracic duct cyst rather than a lipoma. Dissection around the thoracic duct cyst was performed using a vessel-sealing system to prevent leakage of the chyle, and reliable clipping was performed to resect the cisterna chyli. Histopathological examination revealed smooth muscle structures around the cyst, suggestive of a thoracic duct cyst. The diagnosis of a thoracic duct cyst was made based on a high triglyceride level of 1310 mg/dL on examination of the milky-white cyst fluid. The patient's postoperative course was uneventful, and he was discharged 4 days postoperatively. A CT scan performed 13 months after surgery showed no recurrence.

**Conclusions:**

A rare thoracic duct cyst extending from the mediastinum to the cisterna chyli was safely and completely resected using bilateral thoracoscopic surgery, with the patient in the prone position.

## Background

Thoracic duct cysts can occur anywhere along the pathway from the abdominal cisterna chyli to the subclavian and internal jugular veins of the neck [1]. Mediastinal thoracic duct cysts are rare, with only scattered cases reported in the past [1–39]. According to a national tally of mediastinal tumors in Japan, two out of 4098 (0.05%) cases have been reported, and five out of 921 (0.54%) autopsy cases have been reported [40, 41]. Owing to the rarity of the disease, its diagnosis is difficult, and there are no established treatment guidelines. Here, we report a case of a thoracic duct cyst extending from the caudal aspect of the left main bronchus to the left renal artery that was safely and completely resected via bilateral thoracoscopic surgery in the prone position.

## Case presentation

**Fig. 1:**
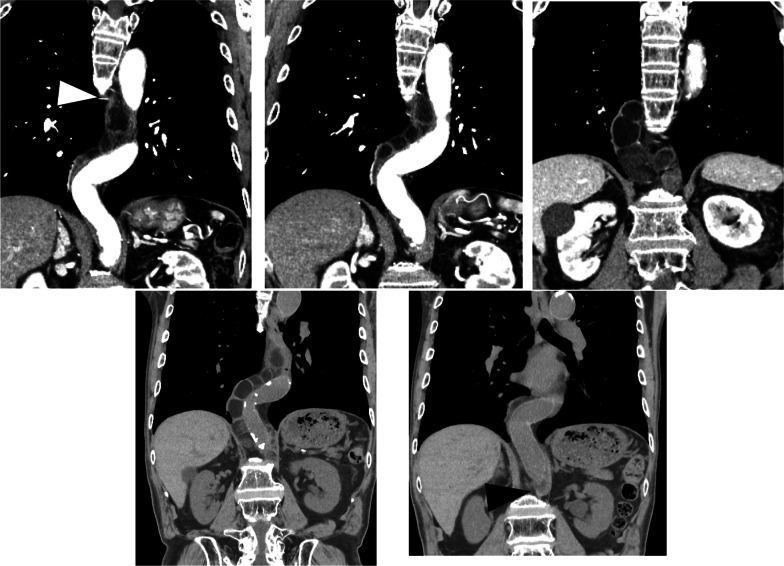
Computed tomography scan showing a continuous multifocal cystic lesion in the cephalocaudal direction from the posterior mediastinum to the retroperitoneum at the 8th thoracic to the 2nd lumbar levels. The white arrowhead (top left) indicates the most cephalad, and the black arrowhead (bottom right) indicates the most caudal

**Fig. 2:**
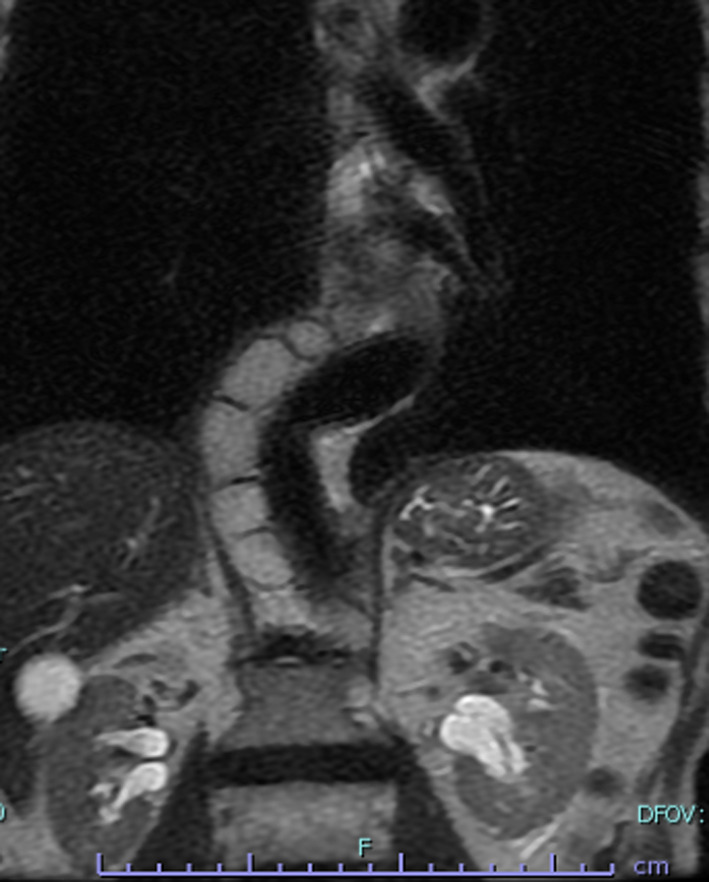
Magnetic resonance imaging showing high intensity on T2-weighted image

**Fig. 3:**
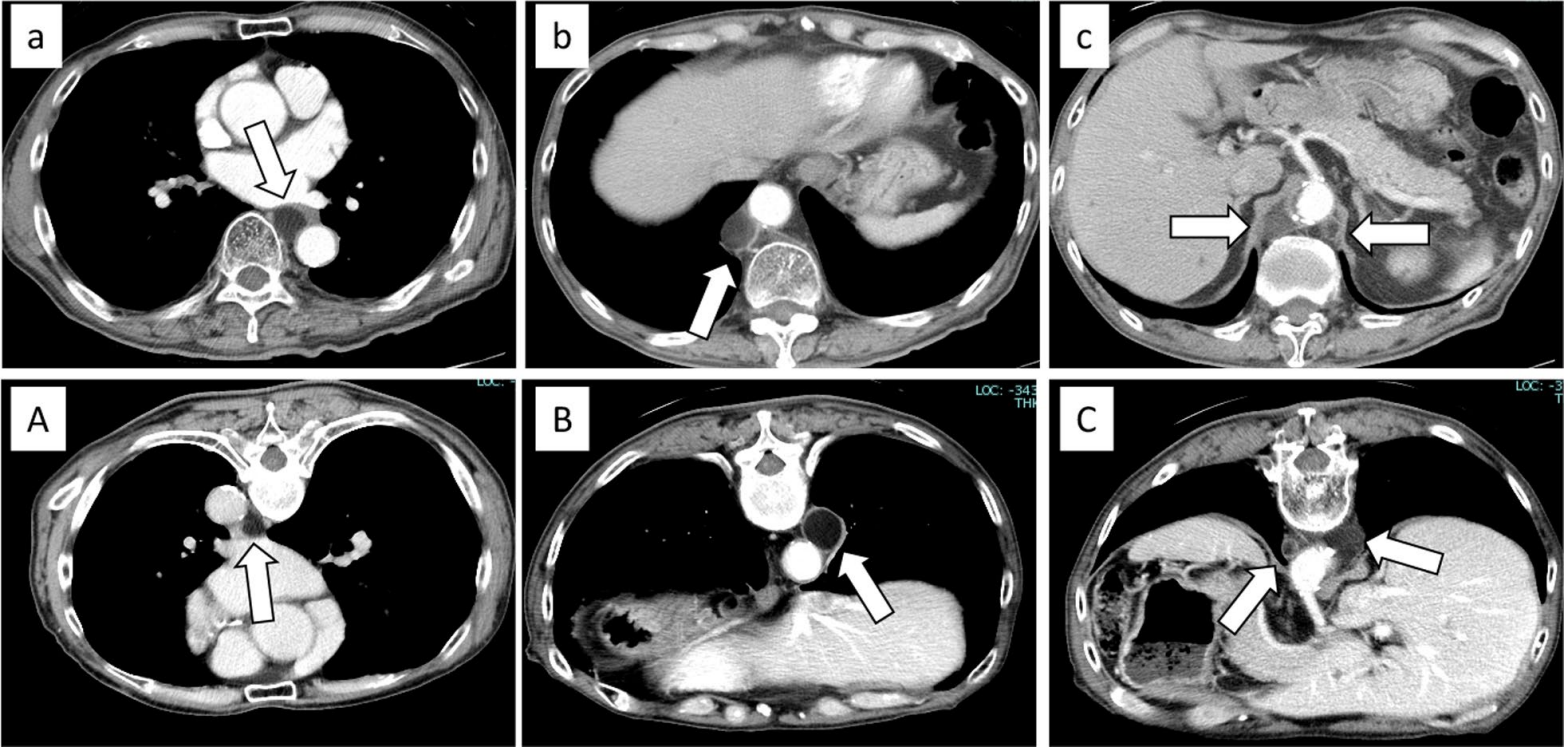
Contrast-enhanced computed tomography in the supine position in the upper row and in the prone position in the lower row. Comparison of **a**, **A** the most cephalic side of the tumor, **b**, **B** confluence of the hepatic vein and inferior vena cava, and **c**, **C** level at which the celiac artery branches off the aorta (white arrow: thoracic duct cyst)

**Fig. 4:**
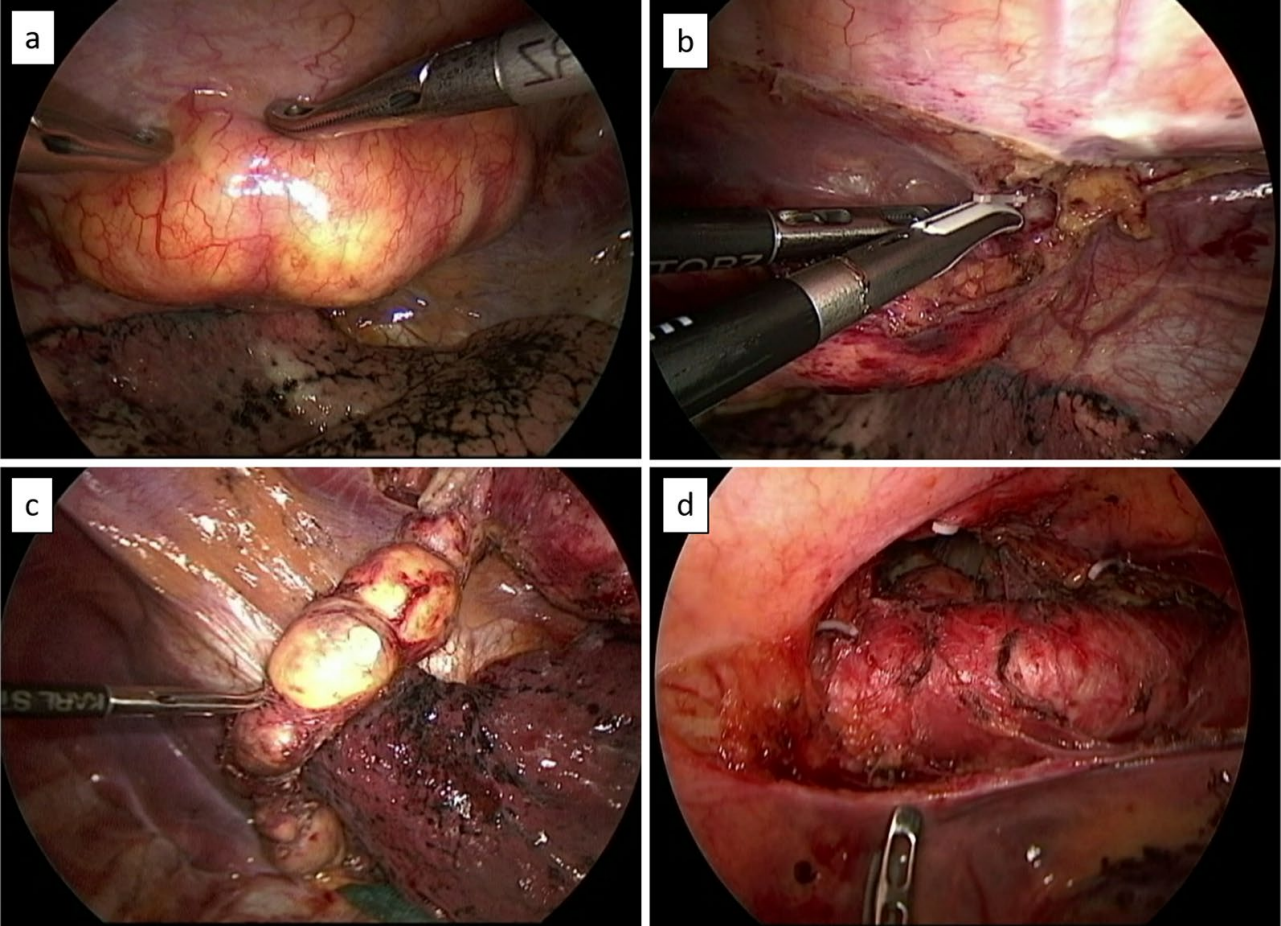
**a** In the right thoracic cavity, the tumor is continuous from the caudal side of the tracheal bifurcation to the diaphragm. **b** To prevent chyle leakage, a vessel-sealing system is used to dissect the tumor perimeter, and the cord-like material is securely clipped and detached. **c** Tumor is multifocal and connected in a bead-like pattern. **d** Left side of the aortic hiatus is opened from the left thoracic cavity, the tumor is dissected from the aorta, and the caudal margin of the tumor is reached. At the caudal margin, clipping is performed, and the dissected specimen is removed

**Fig. 5:**
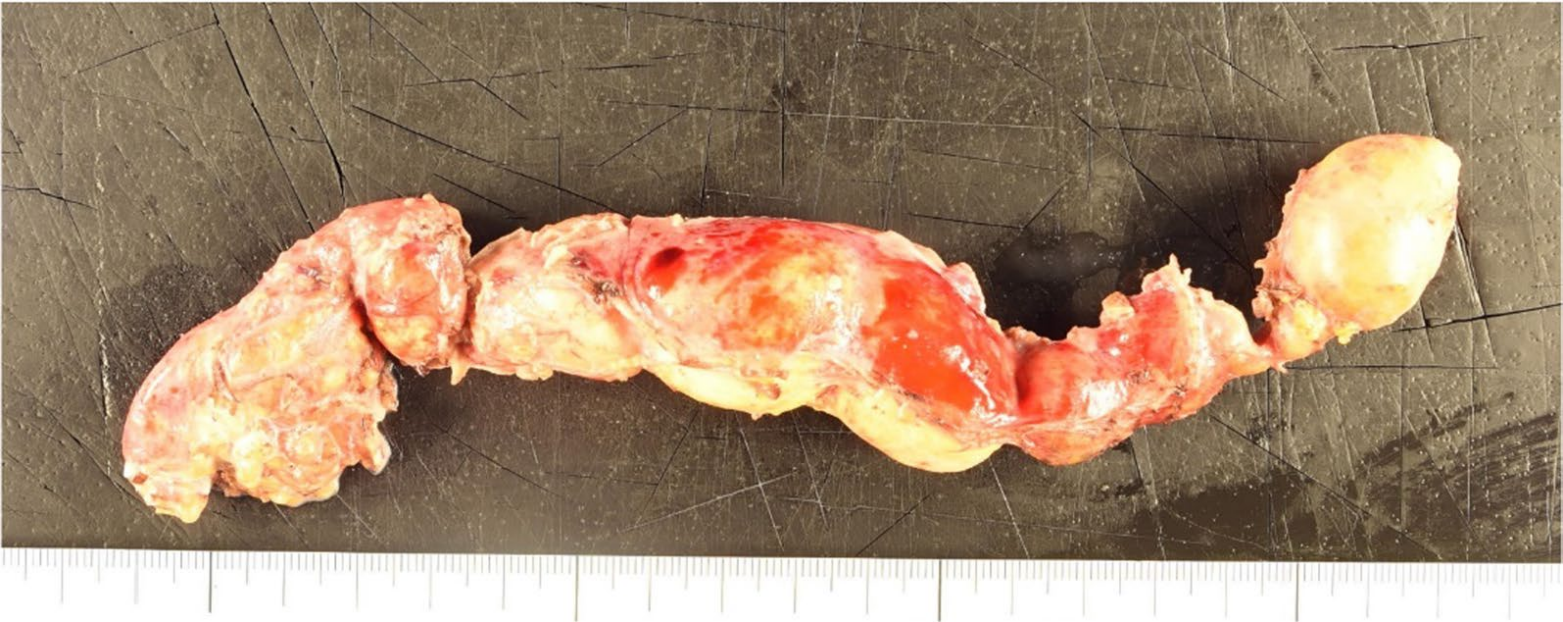
Segmental tumor approximately 20 × 180 mm in size

**Fig. 6:**
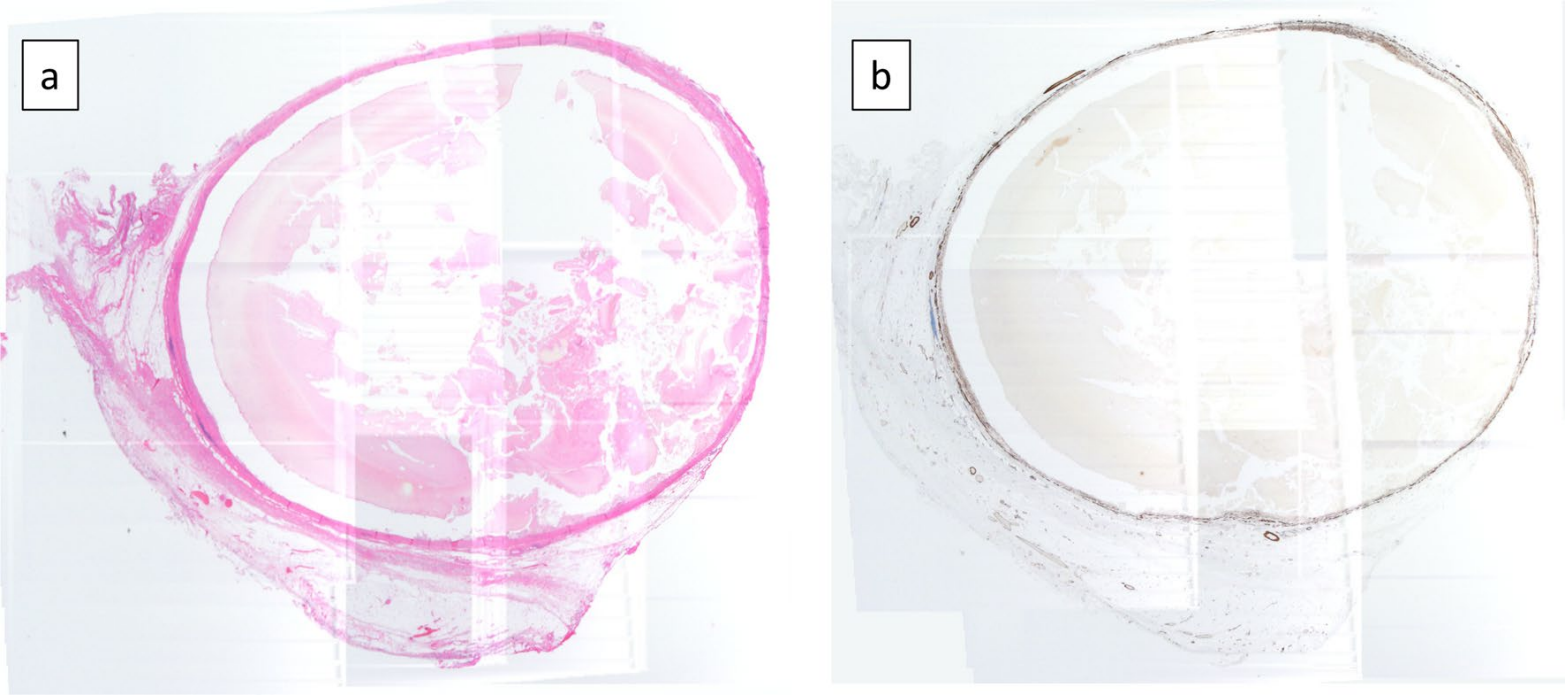
**a** Loupe image of hematoxylin and eosin staining. Cystic structures with fibrous walls are present. **b** Loupe image of α-smooth muscle actin (α-SMA) staining. Smooth muscle stained with α-SMA around the cystic structures

A 77-year-old male patient was referred to our hospital after a computed tomography (CT) scan performed during a follow-up after prostate cancer surgery at another hospital revealed a gradually enlarging mediastinal tumor. He had a history of hypertension, no recurrence after prostate cancer surgery, right inguinal hernia surgery, no medications, occasional drinking, and smoking 20 cigarettes per day. Blood test results revealed the following: total cholesterol, 283 mg/dL; low-density lipoprotein cholesterol, 191 mg/dL; triglyceride (TG), 44 mg/dL, tumor-related marker carcinoembryonic antigen, 6.2 ng/mL; alpha-fetoprotein, 3.9 ng/mL; squamous cell carcinoma antigen, 1.9 ng/mL; cytokeratin 19 fragment, 4.5 ng/mL; soluble interleukin-2 receptor, 429 U/mL; pro-gastrin releasing peptide, 63.9 pg/mL; thyroid stimulating hormone, 1.770 μIU/mL; and human chorionic gonadotropin beta subunit, ≤ 0.1 ng/mL. Contrast-enhanced CT revealed a cephalocaudally continuous multifocal cystic lesion from the posterior mediastinum to the retroperitoneum at the level of the 8th thoracic vertebra to the 2nd lumbar vertebra. The tumor was approximately 20 mm in maximum size, tortuous throughout, but about 180 mm in linear distance, with a predominantly fatty component inside, septal and partly contrast-enhanced components (Fig. 1). Magnetic resonance imaging revealed a high-intensity T2-weighted image, which led to a radiological diagnosis of lipoma or well-differentiated liposarcoma (Fig. 2). In addition, CT in the prone position showed that gravity widened the thoracic cavity, allowing resection from the thoracic cavity to the caudal side of the tumor; therefore, we decided to perform bilateral thoracoscopic surgery in the prone position (Fig. 3). The patient underwent surgery in the prone position, with surgical monitors placed on the left, right, and caudal sides. Because the head end of the tumor was on the right side, a right thoracoscopic surgery was initiated. We placed a 12-mm port in the 7th intercostal space above the posterior axillary line, a 5-mm port in the 9th intercostal space, and a 12-mm port (camera) in the 8th intercostal space above the inferior scapular angle line. The tumor was continuous from the caudal side of the tracheal bifurcation beyond the diaphragm. The intraoperative diagnosis of thoracic duct cyst was made based on the presence of a thicker membrane structure than that of lipoma and hyperdifferentiated liposarcoma, firmer palpation with forceps, and the fact that it coincided with the location of the thoracic duct often seen during esophageal surgery. To prevent chyle leakage, a vessel-sealing system was used to dissect the area around the tumor, and inflow vessels were clipped as needed. All intercostal arteries adjacent to the tumor were preserved to maintain the blood flow to the Adamkiewicz artery. The right side of the diaphragmatic leg aortic hiatus was opened, and the tumor was dissected circumferentially to the point, where it continued to the left side of the aorta, completing the approach from the right thoracic cavity. The tumor was mostly located on the right side of the mediastinum; however, the most caudal side of the tumor in the abdomen was on the left side of the abdominal aorta. The right-sided approach had a poor view of the most caudal side, and we thought that it was necessary to add a left-sided approach due to ensure a better view of the most caudal side of the tumor for reliable clipping. Next, we performed a left thoracoscopic surgery. A 5-mm port was placed in the 7th intercostal space above the left midaxillary line, a 12-mm port in the 9th intercostal space, and a 12-mm port (camera) in the 8th intercostal space above the inferior angle of the scapula. The left side of the diaphragmatic leg aortic hiatus was opened, and the tumor was detached from the vertebral body and aorta to reach the caudal margin. At the caudal margin, the patient was clipped to the contiguous left cisterna chyli, and the dissected specimen was removed. A drain was placed at the midaxillary line of the 9th intercostal space on the patient's right side, and the surgery was completed (Fig. 4). The operation lasted 4 h 30 min, and there was minimal blood loss. The excised specimen was a segmental tumor approximately 20 × 180 mm in size, and the surgical findings were suggestive of a thoracic duct cyst rather than a lipoma (Fig. 5). Histopathological examination revealed a cystic structure with a fibrous wall and smooth muscle stained with alpha-smooth muscle actin around the cystic structure (Fig. 6). The tumor appears to be segmented; however, the interior of the tumor was highly degenerated and no valves inherent to the thoracic duct were histopathologically observed. The cystic fluid was milky white with a high TG level of 1310 mg/dL. Based on these findings, a diagnosis of a thoracic duct cyst was made. The patient's postoperative course was uneventful, and he was discharged 4 days postoperatively. A CT performed 13 months after surgery showed no recurrence.

## Discussion

Thoracic duct cysts are thought to be caused by congenital fragility of the thoracic duct wall as well as degeneration and lymphangiomatous changes secondary to infection and inflammation [1]. Thoracic duct cysts can occur above or below the diaphragm; supradiaphragmatic thoracic duct cysts are usually found in the neck. Mediastinal cysts comprise bronchogenic cysts or esophageal duplication cysts, and mediastinal thoracic duct cysts are rare [23] and only scattered cases have been reported in the past. In reviewing previous reports, we found 39 cases reported. Table 1 shows previous 39 reports of mediastinal thoracic duct cysts and our current case. Of these, three cases including the present case, showed tumors in the retroperitoneum, and the present case was the only one in which surgery was performed thoracoscopically [1–39]. Therefore, there are no established treatment guidelines, and a definitive preoperative diagnosis is difficult. Although the anatomical location varies, a diagnosis can be made using imaging studies, such as CT and magnetic resonance imaging, lymphatic scintigraphy, and lymphangiography [12, 13]. One clinical feature of the disease is that it is often asymptomatic; however, chest pain and dysphagia may occur, and these symptoms may worsen after eating. This is thought to be due to an increase in chyle in the chest duct caused by eating [42]. The TG level of the cyst contents is usually high and was also high in this case (1310 mg/dL). A definitive diagnosis requires histological examination to confirm the presence of the cyst structure and the surrounding smooth muscle cells and lymphatic endothelium. In this case, endothelial cells were lost, and no obvious lymphatic endothelium was detected on D2–40 staining. However, the diagnosis of thoracic duct cysts was made based on the presence of smooth muscle structures around the cystic structures, milky white contents with high TG levels, and intraoperative findings. Surgery is the only treatment method, but since it is a benign disease, follow-up observation is an option if it is asymptomatic; however, since rupture cases have been reported, surgery is considered if there is a tendency toward enlargement [38]. In this case, surgery was performed, because a malignant tumor was suspected; however, even if the preoperative diagnosis would have been a thoracic duct cyst, surgery seemed to prevent further enlargement. Because prevention of chyle leakage is important for the resection of thoracic duct cysts, all vascular structures, such as the collecting ducts that flow into the thoracic duct cyst, must be clipped or ligated and dissected without failure. As mentioned above, it is difficult to make a definitive diagnosis of a thoracic duct cyst before surgery, but it is important to always keep in mind the possibility of a thoracic duct cyst if a mediastinal tumor is diagnosed, because tumor removal without ligation of the duct without suspicion of the possibility of a thoracic duct cyst can lead to the development of chyle leakage. When a tumor is located caudally from the inferior mediastinum, it is important to be aware of the blood flow to the Adamkiewicz artery, preserve the vessels around the tumor as much as possible, and search for the Adamkiewicz artery on CT before surgery. The intercostal arteries were adjacent to the tumor on preoperative CT. It is necessary that careful manipulation should be performed to avoid injury to the intercostal artery, such as Adamkiewicz artery. In this case, the patient was not obese, with a body mass index of 19.1. Moreover, the CT scan in the prone position showed that the thoracic cavity was widened by gravity. Thus, it was judged that it was possible to resect from the thoracic cavity to the caudal side of the tumor. However, it is questionable whether the diaphragm is elevated in patients with obesity and whether the prone position is useful. The prognosis is considered good, because there have been no reports of recurrence, even in cases where fluid leakage was observed during intraoperative manipulation or in cases of postoperative chyle leakage.

**Table 1:** Summary of case reports of mediastinal thoracic duct cysts since 1950

No	Authors	Year	Sex	Age	Location	Size (mm)	Symptoms
1	Emerson [2]	1950	F	20	Middle mediastinum	40 × 55	Substernal pain, dyspnea, cough
2	Bakst [3]	1954	M	42	Lower mediastinum	60 × 80	Epigastric pain
3	Cohen [4]	1962	M	35	−	−	Chest pain
4	Thomas [5]	1966	M	43	−	−	Asymptomatic
5	Beasley [6]	1971	F	46	−	−	Asymptomatic
6	Fromang [7]	1975	F	35	Posterior mediastinum	−	Respiratory insufficiency, superior vena cava compression
7	Cervantes-Peres [8]	1976	M	42	−	−	Postprandial retrosternal pain
8	Gowar [9]	1978	F	45	Superior mediastinum	60 × 40	Dyspnea
9	Luosto [10]	1978	F	56	−	−	Asymptomatic
10	Den Otter [11]	1979	M	60	Anterior mediastinum	−	Asymptomatic
11	Tsuchiya [12]	1980	F	49	−	−	Eructation
12	Hori [13]	1980	M	40	−	−	Retrosternal pain, dysphagia, dyspnea
13	Morettin [14]	1986	M	57	−	−	Asymptomatic
14	Adachi [15]	1989	F	27	Posterior mediastinum	−	Asymptomatic
15	Takahashi [16]	1990	M	72	Superior mediastinum	75 × 45 × 45	Dysphagia
16	Mori [17]	1992	M	86	Middle mediastinum	100 × 50	Dyspnea, cyanosis
17	Muramatsu [18]	1992	M	33	Superior mediastinum	−	Asymptomatic
18	Okabe [19]	1993	M	45	−	60	Asymptomatic
19	Lamers [20]	1994	M	57	−	−	Retrosternal pain, cough
20	Nishizaki [21]	1996	M	55	Posterior lower mediastinum	20 × 30 × 60	Asymptomatic
21	Chen [22]	1999	M	34	Superior mediastinum	30 × 50 × 150	Asymptomatic
22	Karajiannis [23]	2000	M	49	Anterior superior mediastinum	80 × 100 × 120	Asymptomatic
23	Suzuki [24]	2001	F	34	Superior mediastinum	65 × 40	Left supraclavicular mass
24	Pramesh [25]	2003	M	49	Superior mediastinum	−	Hoarseness
25	Turkyilmaz [26]	2007	F	82	Posterior lower mediastinum	90 × 100	Dysphagia
26	Matwiyoff [27]	2008	M	28	Lower mediastinum	25 × 15	Chest pain
27	Mortman [1]	2009	F	68	Posterior lower mediastinum	59 × 67 × 72	Nonproductive cough, mild dyspnea on exertion, and chest pressure
28	De Santis [28]	2010	F	30	Superior mediastinum	90 × 70	Dry cough and hiccups
29	Kwak [29]	2011	F	53	Superior mediastinum	−	Asymptomatic
30	Taniguchi [30]	2011	M	29	Middle mediastinum	−	Asymptomatic
31	Wada [31]	2012	F	58	Middle mediastinum	40 × 20 × 10	Asymptomatic
32	Park [32]	2015	F	42	Posterior lower mediastinum	−	Asymptomatic
33	Wan [33]	2015	M	35	Posterior mediastinum to retroperitoneum	42 × 50 × 114	Abdominal pain
34	Halliday [34]	2015	M	66	Superior mediastinum	20	Hoarseness
35	Electra [35]	2016	F	28	Posterior lower mediastinum	45 × 35 × 8	Asymptomatic
36	Kamata [36]	2017	M	54	Superior mediastinum	−	Nausea and hypotension
37	Abu-Zaid [37]	2018	M	57	Posterior mediastinum to retroperitoneum	130	Right upper quadrant pain
38	Garner [38]	2020	F	48	Posterior middle mediastinum	130 × 90	Abdominal pain on a background of exertional dyspnoea and orthopnoea
39	Cabral [39]	2022	M	74	Lower mediastinum	17	Asymptomatic
40	Present case	2023	M	77	Posterior middle mediastinum—retroperitoneum	180 × 20	Asymptomatic

## Conclusions

We report a rare case of a thoracic duct cyst extending from the mediastinum to the cisterna chyli. Complete resection was safely achieved using bilateral thoracoscopic surgery, with the patient in the prone position.

## Abbreviations

CT: Computed tomography
TG: Triglyceride

## References

[1] Mortman KD (2009). Mediastinal thoracic duct cyst. Ann Thorac Surg.

[2] Emerson GL (1950). Supradiaphragmatic thoracic-duct cyst. An unusual mediastinal tumor. N Engl J Med.

[3] Bakst AA (1954). Blind supradiaphragmatic thoracic duct cyst; case report. Ann Surg.

[4] Cohen EB, Kompaniez E (1962). Supradiaphragmatic thoracicduct cyst. Report of a case. N Engl J Med.

[5] Thomas MJ, Sanger PW, Taylor FH, Robicsek F (1963). Thoracic duct cyst of the mediastinum; a case report. Coll Works Cardiopulm Dis.

[6] Beasley WE, Mills M (1971). Chylous cystectomy with prosthetic reconstruction of the diaphragm and esophageal hiatus. J Thorac Cardiovasc Surg.

[7] Fromang DR, Seltzer MB, Tobias JA (1975). Thoracic duct cyst causing mediastinal compression and acute respiratory insufficiency. Chest.

[8] Cervantes Perez P, Fuentes-Maldonado R (1976). Thoracic duct cyst of the mediastinum. Chest.

[9] Gowar FJ (1978). Mediastinal thoracic duct cyst. Thorax.

[10] Luosto R, Koikkalainen K, Jyrälä A, Mäkinen J (1978). Thoracic duct cyst of the mediastinum: a case report. Scand J Thorac Cardiovasc Surg.

[11] Den Notter G (1979). Thoracic duct cyst in the anterior mediastinum. Arch Chir Neerl.

[12] Tsuchiya R, Sugiura Y, Ogata T, Suemasu K (1980). Thoracic duct cyst of the mediastinum. J Thorac Cardiovasc Surg.

[13] Hori S, Harada K, Morimoto S, Uchida H, Okumura K (1980). Lymphangiographic demonstration of thoracic duct cyst. Chest.

[14] Morettin LB, Allen TE (1986). Thoracic duct cyst: diagnosis with needle aspiration. Radiology.

[15] Adachi A, Watanabe J, Kurohiji T, Nishimura Y, Edakuni S, Kakegawa T (1989). A case report of thoracic duct cyst. Nihon Kyobu Geka Gakkai Zasshi.

[16] Takahashi C, Hanyuda M, Fukaya Y, Nohara H, Morimoto M, Iida F (1990). Thoracic duct cyst–a case report. Nihon Kyobu Geka Gakkai Zasshi.

[17] Mori M, Kidogawa H, Isoshima K (1992). Thoracic duct cyst in the mediastinum. Thorax.

[18] Muramatsu M, Tamura N, Doi Y, Dambara T, Uekusa T, Masuda S, Nukiwa T, Kira S (1992). A case of mediastinal lymphatic cyst possibly originating from the right thoracic duct. Nihon Kyobu Shikkan Gakkai Zasshi.

[19] Okabe K, Miura K, Konishi H, Hara K, Shimizu N (1993). Thoracic duct cyst of the mediastinum: case report. Scand J Thorac Cardiovasc Surg.

[20] Lamers RJ, van Belle AF (1993). Thoracic duct cyst in the middle part of the mediastinum. AJR Am J Roentgenol.

[21] Nishizaki K, Ohno K, Hatanaka N, Yamamoto S, Kuwata K, Kobayashi Y (1996). Mediastinal thoracic duct cyst–a case report. Nihon Kyobu Geka Gakkai Zasshi.

[22] Chen F, Bando T, Hanaoka N, Terada Y, Ike O, Wada H, Hitomi S (1999). Mediastinal thoracic duct cyst. Chest.

[23] Karajiannis A, Krueger T, Stauffer E, Ris HB (2000). Large thoracic duct cyst—a case report and review of the literature. Eur J Cardiothorac Surg.

[24] Suzuki Y, Ogawa N, Mukai K, Ishiwa N (2001). A case of mediastinal thoracic duct cyst. Kyobu Geka.

[25] Pramesh CS, Deshpande MS, Pantvaidya GH, Sharma S, Deshpande RK (2003). Thoracic duct cyst of the mediastinum. Ann Thorac Cardiovasc Surg.

[26] Turkyilmaz A, Eroglu A (2007). A giant thoracic duct cyst: an unusual cause of dysphagia. J Thorac Cardiovasc Surg.

[27] Matwiyoff GN, Bradshaw DA, Hildebrandt KH, Campenot JF, Coletta JM, Coyle WJ (2008). A 28-year-old man with a mediastinal mass. Thoracic duct cyst Chest.

[28] De Santis M, Martins V, Fonseca AL, Santos O (2010). Large mediastinal thoracic duct cyst. Interact Cardiovasc Thorac Surg.

[29] Kwak MY, Bae CH (2011). Thoracic duct cyst in mediastinum—a case report -. Korean J Thorac Cardiovasc Surg.

[30] Taniguchi Y, Miwa K, Adachi Y, Fujioka S, Haruki T, Nakamura H (2011). Thoracoscopic resection of a thoracic duct cyst that developed during follow-up for a thymic cyst. Gen Thorac Cardiovasc Surg.

[31] Wada H, Yoshida S, Ishikawa A, Yasufuku K, Yoshino I, Kimura H (2012). Endobronchial ultrasonography in a patient with a mediastinal thoracic duct cyst. Ann Thorac Surg.

[32] Park SJ, Park SY, Choi H (2015). Aberrant thoracic duct cyst in postrior mediastinum. Korean J Thorac Cardiovasc Surg.

[33] Wan X, Zhou Z (2015). A giant thoracic duct cyst as the cause of abdomen pain: a case report and review of the literature. Ann Thorac Cardiovasc Surg.

[34] Halliday LJ, Sharma AK (2015). Multiple thoracic duct cysts: an unusual CT finding. J Surg Case Rep.

[35] Electra MM, Evangelia A, Mattheos B, Dimitris HI, Zarogoulidis P, Tsavlis D, Kougioumtzi I, Machairiotis N, Charalampidis C, Fassiadis N, Mparmpetakis N, Pavlidis P, Andreas M, Stamatis A, Alexandros K, Kosmas T (2016). Thoracic duct cyst of posterior mediastinum: a "challenging" differential diagnosis. Ann Transl Med.

[36] Kamata T, Shiba M, Fujiwara T, Nagato K, Yoshida S, Inoue T (2017). Chylopericardium following thoracoscopic resection of a mediastinal cyst: a case report. Int J Surg Case Rep.

[37] Abu-Zaid A, Alakhtar AM, Alshamdin FD, Saleh W (2018). Thoracic duct cyst presenting as abdominal pain. Surgery.

[38] Garner M, Duvva D, Gosney J, Buderi S (2020). Spontaneousrupture of a giant thoracic duct cyst presenting with abdominal pain and a tension chylothorax. Interact Cardiovasc Thorac Surg.

[39] Cabral SM, Matos P, Santis M (2022). Mediastinal thoracic duct cyst—an unusual finding. Arch Bronconeumol.

[40] Masaoka A, Yamaguchi S, Mori T (1971). National collection of vertical septal surgery. Jpn J Thorac Surg.

[41] Kasai M, Terasawa Y (1964). Frequency and pathology of mediastinal tumours. Thorac Dis.

[42] Sato S, Hanzawa T, Hada T, Tsuchiya K, Miyoshi I, Itsubo K (1993). Mediastinal thoracic duct cyst; a case report. J Jpn Assoc Chest Surg.

